# The diagnostic accuracy of carbon monoxide pulse oximetry in adults with suspected acute carbon monoxide poisoning: a systematic review and meta-analysis

**DOI:** 10.3389/fmed.2023.1250845

**Published:** 2023-12-28

**Authors:** Giacomo Ramponi, Francesca Gianni, Eleni Karlafti, Isabelle Piazza, Francesco Albertoni, Giorgio Colombo, Giovanni Casazza, Anna Garegnani, Rosa Casella, Giorgio Costantino

**Affiliations:** ^1^Dipartimento di Scienze Cliniche e Comunità, Università degli Studi di Milano, Milan, Italy; ^2^Pronto Soccorso, Dipartimento di Emergenza Urgenza, Fondazione IRCCS Ca’ Granda Ospedale Maggiore Policlinico, Milan, Italy; ^3^Emergency Department, AHEPA University Hospital, Aristotle University of Thessaloniki, Thessaloniki, Greece; ^4^Pronto Soccorso, ASST Papa Giovanni XXIII, Bergamo, Italy; ^5^Retired, Latina, Italy

**Keywords:** carbon monoxide, carbon monoxide poisoning, pulse oximetry, diagnostic accuracy, carboxyhemoglobin, oximetry, intoxication, systematic review & meta-analysis

## Abstract

**Introduction:**

Acute carbon monoxide poisoning (COP) is one of the leading causes of intoxication among patients presenting to the emergency department (ED). COP symptoms are not always specific and may vary from mild to critical. In the last few years, COHb pulse oximeters have been developed and applied to the setting of suspected COP. The aim of this systematic review is to assess the diagnostic accuracy of CO pulse oximetry (SpCO) with carboxyhemoglobin (COHb) levels measured by blood gas analysis, used as a reference standard, in patients with suspected COP.

**Methods:**

We developed our search strategy according to the PICOS framework, population, index/intervention, comparison, outcome, and study, considering the diagnostic accuracy of SpCO compared to COHb levels measured by blood gas analysis, used as a reference standard, in patients with suspected COP enrolled in cross-sectional studies in English. The search was performed on MEDLINE/PubMed and EMBASE in February 2022. Quality assessment was performed using the QUADAS-2 methodology. A COHb cutoff of 10% was chosen to test the sensitivity and specificity of the index test. A bivariate model was used to perform the meta-analysis. The protocol was registered on PROSPERO (CRD42022359144).

**Results:**

A total of six studies (1734 patients) were included. The pooled sensitivity of the test was 0.65 (95% CI 0.44–0.81), and the pooled specificity was 0.93 (95% CI 0.83–0.98). The pooled LR+ was 9.4 (95% CI 4.4 to 20.1), and the pooled LR- was 0.38 (95% CI 0.24 to 0.62).

**Conclusion:**

Our results show that SpCO cannot be used as a screening tool for COP in the ED due to its low sensitivity. Because of its high LR+, it would be interesting to evaluate, if SpCO could have a role in the prehospital setting as a tool to quickly identify COP patients and prioritize their transport to specialized hospitals on larger samples with a prospective design.

## Background

Carbon monoxide (CO) is a colorless, odorless, non-irritant, toxic gas. The incomplete combustion of hydrocarbons leads to CO production. In nature, the principal CO sources are volcanoes and forest fires (1, 2). On the other hand, artificial CO production comes mainly from motor vehicles, poorly functioning heating systems, gas-powered equipment, house fires, boat engines, and cigar or waterpipe smoking. These factors contribute to most cases of accidental human CO poisoning (COP) (3). CO intoxication can also result from indirect sources, such as dichloromethane ingestion or inhalation and its subsequent metabolism to CO (4).

CO intoxication is one of the leading poisoning causes of admission to the ED, with an exposure that may be accidental or intentional (3, 5). According to European registries on CO intoxications and deaths, from 1980 to 2008, there were 140,490 CO-related deaths in 28 countries. CO intoxication was involved in 31,473 hospital admissions. Death due to unintentional intoxication (mostly caused by accidental exposure, such as that due to faulty heating systems and appliances) accounts for 54.7% of all CO-related deaths (6). Intentional COP can take place by several means, such as self-intoxication with internal combustion engine exhaust fumes in an enclosed space.

CO is inhaled and then diffuses from the alveoli into the blood, with its blood levels depending upon its concentration in the air and the duration of exposure (1). After absorption, CO partially binds to myoglobin and cytochrome C, but mainly to iron molecules of hemoglobin, so that it forms carboxyhemoglobin (COHb) (1, 5, 7). Hemoglobin affinity for CO is up to 250 times higher than oxygen affinity (1, 5). This implies that oxygen delivery to tissues is severely impaired, and hypoxia and anaerobic metabolism result as a consequence (1, 3). In addition, CO affects platelet function and may lead to a hypercoagulable state and intravascular thrombosis (8). Furthermore, oxidative metabolism is affected by CO and leads to free radical formation (9).

COP symptoms are not always specific and have a wide range, from mild to critical (5, 9). Mild symptoms such as headache, nausea, dizziness, and vomiting may be present. In more severe cases, patients may present with syncope or altered mental status. Moreover, CO may cause cardiac ischemia or failure due to hypoxia and endothelial dysfunction of myocytes in combination with mitochondrial inhibition, promoting a syndrome similar to myocardial infarction (5, 9).

Since the diagnosis of COP is complicated by the variability in its clinical presentations, it can sometimes go undetected (10). Consequently, the absence of a clear history and the non-specific symptoms make blood CO-oximetry a fundamental adjunct to confirm or exclude a suspected exposure in different settings. Blood gas analysis (BGA) is the most widespread method for the diagnosis of COP because the measurement of COHb levels is reliable and quickly available. Nonetheless, BGA is an invasive method and is usually not available out of the hospital, in particular, in emergency situations with contingent severity and an urgent need for therapy (7, 10). Since standard pulse oximetry devices are not able to distinguish between oxyhemoglobin and COHb, oxygen saturation levels in patients with COP are wrongly reported as normal (11). Since 2005, the Rad-57 signal extraction pulse CO-oximeter (RAD; Maximo Corporation, Irvine, CA) has been approved for clinical use. This device has the ability to discriminate between oxyhemoglobin, COHb, and methemoglobin and therefore may be used to assess the COHb percentage level. Several studies have been published addressing this issue, with partially discordant results (11, 12).

The aim of this systematic review is to evaluate the diagnostic accuracy of CO-oximetry (SpCO) using blood COHb as a reference standard in adult patients with suspected COP.

## Methods

This systematic review was conducted and reported in accordance with the Preferred Reporting Items for Systematic Reviews and Meta-Analyses (PRISMA) statement. The protocol of this systematic review was registered on PROSPERO with the registration number CRD42022359144.

### Inclusion criteria

Studies that enrolled patients suspected of COP, published in English, with a cross-sectional design were eligible if data to construct a 2×2 table for SpCO accuracy at a cutoff of 10% were available. The 10% cutoff was chosen because it is outside of the physiological range for heavy smokers. The index test had to be peripheral SpCO and the reference standard COHb measured by BGA.

### Search strategy and screening for inclusion

We developed our search strategy according to the PICOS framework, population, index/intervention, comparison, outcome, and study, considering the diagnostic accuracy of SpCO compared to COHb levels measured by BGA in patients with suspected COP enrolled in cross-sectional studies in English.

MEDLINE/PubMed and EMBASE were searched for cross-sectional studies in February 2022. The search strategy was ((diagnosis) OR (sensitivity) OR (specificity)) OR (cross-sectional) AND (carbon monoxide).

Reference lists of individual articles were manually screened for evaluation and possible inclusion of other relevant studies.

After removing duplicates, abstracts were independently screened by two review authors (EK and FA). Full texts of the potentially eligible studies were retrieved, and two review authors (GR and FA) independently assessed the full-text publications for eligibility according to inclusion criteria. Disagreements between investigators in the choice of article inclusion (such as those due to the relevance of the article to the research question, study design, and patient selection criteria) were resolved by means of inter-investigator discussion and eventual agreement. When disagreement persisted, a third investigator (GC) was asked to make the eventual decision.

### Data extraction and quality assessment

The following data were extracted: study title, author, country, design, language and year of publication, number of centers involved, funding, the total number of patients, age range, sex, and data for a 2×2 table (numbers of true positive, TN; false positive, FP; false negative, FN; and true negative, TN) at a cutoff of 10%. For studies that did not report in the main text or tables, accuracy data and data for the 2×2 table were derived from figures reported in the article, if available.

The Quality Assessment of Diagnostic Accuracy Studies (QUADAS-2) tool was used for the assessment of the risk of bias in the included studies and the applicability of their results.

All data were extracted by two review authors (FA and GR). Disagreements were resolved through discussion.

### Statistical analysis

First, we performed a graphical descriptive analysis of the included studies. We presented forest plots [sensitivity and specificity separately, with their 95% confidence intervals (CIs)], and we provided a graphical representation of studies in the receiver operating characteristic space (sensitivity against 1 – specificity). Second, we performed the meta-analyses using the bivariate model and provided estimates of pooled sensitivity and specificity. We used the summary pooled estimates obtained from the fitted models to calculate summary estimates of positive (LR+) and negative (LR–) likelihood ratios.

All the statistical analyses were performed using SAS (release 9.4) and Rev Man (release 5.4) software.

## Results

### Study selection

**Figure 1:**
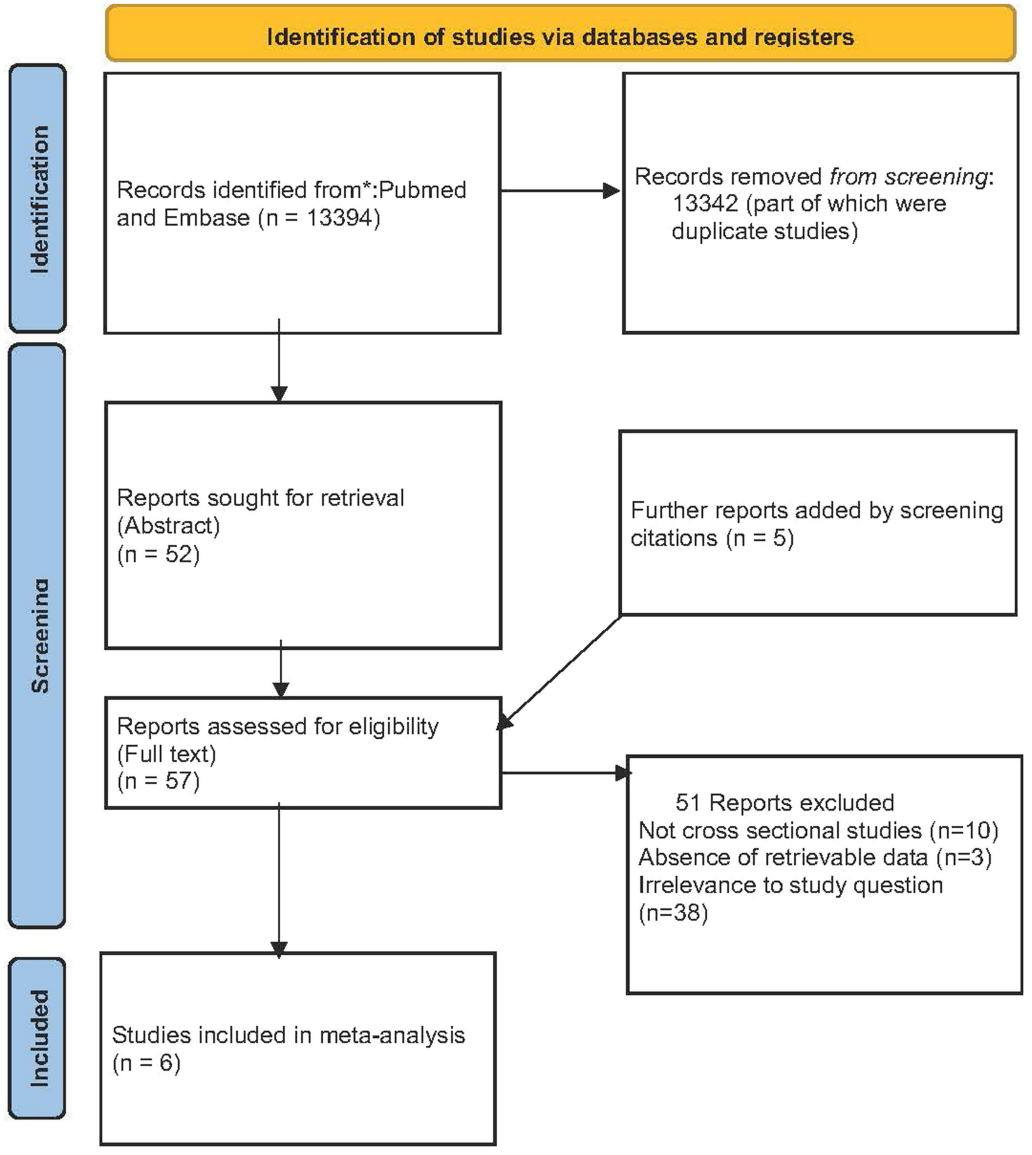
Flow diagram of study selection.

The search, after removing duplicates, yielded 13,394 results, of which 13,342 were excluded after screening the title or the abstract of the studies. The remaining 52 studies were assessed for inclusion. Five additional studies were included after the manual screening of the 52 full-text references and were also read in full. Out of the 57 articles evaluated for full assessment, 38 studies were not relevant to the study question, 10 studies were not cross-sectional, and data could not be retrieved for three additional studies. Finally, six studies were included in the systematic review and meta-analysis (7, 10, 11, 13–15). The study selection process is summarized in Figure 1.

### Characteristics of the included studies

All the studies included were monocentric, with prospective data collection conducted from 2005 to 2014. A total of 1734 patients were included. The mean age was in the fifth decade for most of the studies. Females accounted for 40–55% of the subjects. The number of participants for each study varies from 12 to 1,363. Smoking habits were reported in three out of six studies (11, 13, 14) and smokers percentages ranged from 23 to 40%. Out of 1734 patients, 106 had COP. The overall prevalence of COP was 6.1%, ranging from 1.7% (in the largest study) to 83% (in the smallest study). Pediatric patients were only present in Piatkowski’s study (one patient, age 8), in Coulange’s study (one patient, age 8), in Weaver’s study (at least one patient, age 3), and possibly in Touger’s study (not specified). The characteristics of the included studies are presented in Table 1.

**Table 1:** Characteristics of studies included within the systematic review.

Study primary reference	Publication year	Dates of study beginning and ending	Country	Initial population of interest	Study design	Number of centers	Funding and competing interests	Index test	Test methodology	Reference standard	Number of participants	Age (Mean ± SD or median with interquartiles)	Male percent	Dropout/ NA data / excluded patients	Inclusion criteria for study entry	Exclusion criteria for study entry	Primary outcome	Secondary outcomes	Diagnostic cutoffs
Coulange et al. (13)	2008	October 2005 to April 2006	France	Patients (adults and children) admitted to the Sainte Marguerite Hospital in Marseille ED with suspected CO poisoning	Observational study, cross-sectional design (prospective data collection)	1	This prospective descriptive study was undertaken independently, with no funding from the device manufacturer.	Non-invasive SpCO analysis using pulse CO oximetry (Rad57, Masimo Corp., USA)	Simultaneous measurement	Spectrophotometric measurement on a venous blood sample (IL 682 CO-oximeter, Instrumentation Laboratory, Barcelona, Spain)	12 patients	41 ± 17	50%	0	Non-smoker adult and pediatric patients admitted to the ED with suspected COP but prior to blood sampling and hospital admission	Smokers	Alignment of the two methods according to the Bland and Altman procedure		Analysis using the Bland and Altman protocol demonstrated good alignment for both techniques with a bias of −1.5%, suggesting that pulse CO-oximetry slightly overestimated. The analysis using the Passing and Bablok statistical protocol also demonstrated good alignment. As the authors provided individual patients’ data, it is possible to build a 2×2 table for a 10% COHb diagnostic cutoff
Piatkowski et al. (10)	2009	January 2006 to August 2008	Germany	Adult males and females were admitted to the burn unit with CO intoxication in the absence of burns. One child (8, male) was also included.	Observational study, cross-sectional design	1	No funding declared, it appears that Masimo Corp. provided the required Rad57 pulse CO oximeter for this study free of charge	Non-invasive SpCO analysis using pulse CO oximetry (Rad57, Masimo Corp., USA)	Blood gas analysis including COHb testing was performed on the first day, hourly. A standard blood gas analyzer (Radiometer GmbH, ABL700) was used to identify the COHb levels. Venous blood drawn from peripheral veins was used for blood gas analysis. At the same time, SpCO was detected non-invasively using the Rad57 pulse CO oximeter (Masimo)	Venous blood gas analysis (Radiometer GmbH, ABL700) with COHb testing	20 patients	42 ± 21	60%	0	Patients who were admitted by ambulance with CO intoxication but without burn injuries. 5 healthy volunteers (unmatched for age and gender) served as the control group	Burn injuries	Mean error of SpCO measurements with respect to venous blood gas COHb testing	Mean error of venous blood gas COHb testing compared to other devices of the same type within the same department	While not relevant to the study outcomes, the diagnostic cutoff for CO poisoning requiring HBO treatment was COHb 10%
Touger et al. (7)	2010	January 2008 and April 2009	United States	Patients presenting to the adult and pediatric EDs of Jacobi Medical Center, New York	Observational study, cross-sectional design (prospective data collection)	1	Masimo Corporation provided 3 RAD-57 Pulse Co-Oximeters for the duration of the study and training.	Non-invasive SpCO analysis using pulse CO oximetry (Rad57, Masimo Corp., USA)	Simultaneous measurement	Arterial or venous blood was obtained with the first RAD measurement and sent to the hospital laboratory in a heparinized syringe for direct measurement of whole blood carboxyhemoglobin, using co-oximetry (Siemens Rapidlab 1,200 blood gas analyzer).	120 patients	31 (16–48)	55%		Patients with suspected carbon monoxide poisoning and for whom arterial or venous blood carboxyhemoglobin testing was performed in the course of regular clinical care were eligible for inclusion.	Patients with burns involving the digits that precluded proper placement of the device were excluded.	Diagnostic accuracy of SpCO testing for CO poisoning compared with BGA COHb testing (10% COHb cutoff). Method 1: 5% discrepancy in Carboxyhemoglobin Method 2: accuracy of SpCO vs. lab COHb with a 15% cutoff		5% discrepancy in Carboxyhemoglobin (2) Accuracy of SpCO vs. lab COHb with a 15% cutoff
Sebbane et al. (11)	2013	19 months period	France	All patients attending the emergency department of Centre Hospitalier Regional Universitaire Lapeyronie, Montpellier	Observational study, cross-sectional design (prospective data collection)	1	No funding nor conflict of interest declared	Non-invasive SpCO analysis using pulse CO oximetry (Rad57, Masimo Corp., USA)	Venous blood sampling is described as “simultaneous” to pulse oximetry measurement. Mean time elapsed was 19 min (95%CI 10–29 min)	COHb testing within an in-hospital laboratory using an automated CO-oximeter (IL 682, Instrumentation Laboratory, Milan, Italy)	93 patients	43 ± 20	45%	95 eligible patients, two excluded for missing data	Suspected CO poisoning		Diagnostic accuracy of SpCO testing for CO poisoning compared with BGA COHb testing	Optimal cutoff for CO poisoning diagnosis with SpCO use.	The laboratory sample cutoff was COHb >5% in non-smokers and COHb >10% in smokers. On the contrary, the SpCO cutoff was derived from ROC curves (9% for the whole population, 6% for non-smokers, and 9% for smokers).
Weaver et al. (14)	2013	April 2008 to August 2008	United States	All patients attending the emergency department of Intermountain Medical Center, Murray, Utah, had a lithium heparin tube of blood drawn for clinical purposes.	Observational study, cross-sectional design (prospective data collection)	1	The authors have disclosed relationships with SciMetrika and Masimo. This study was supported by a grant from the Centers for Disease Control, through and with additional support by SciMetrika. Masimo provided the oximeters for research use.	Non-invasive SpCO analysis using pulse CO oximetry (Rad57, Masimo Corp., USA)	Of subjects having a lithium heparin tube of blood drawn for clinical purposes, study personnel measured the SpCO with the Rad-57 pulse oximeter. After obtaining the pulse oximetry measurement, the technician withdrew 1 mL of blood from the lithium heparin tube, with a blood gas syringe. This sample was taken to the blood gas laboratory	CO-oximetry (ABL 825, Radiometer, Copenhagen, Denmark)	1,363 unselected patients	48 ± 21	45%				False positive rate among patients screened with SpCO oximeter measured with two methods: -difference between SpCO and COHb higher than 3%-SpCO>6% and COHb <6%	False negative rate among patients screened with SpCO oximeter measured with two methods: -difference between SpCO and COHb higher than −3% SpCO<6% and COHb >6%	False positives were patients with a difference between SpCO and COHb higher than 3%. False negatives were patients with a difference between SpCO and COHb higher than −3%. It was possible to infer data about the 2×2 table from the narrative description of the results.
Villalba et al. (15)	2019	June 2011 to March 2014	United States	All patients attending the emergency department of Larner College of Medicine, University of Vermont, Burlington	Observational study, cross-sectional design (prospective data collection)	1	This was an investigator-initiated study supported by the manufacturer of the pulse oximeter (Masimo).	SpCO measurement with Masimo Radical 7 CO oximeters (Masimo Inc., Irvine, CA)	Both tests were performed within 5 min of each other.	COHb testing within an in-hospital laboratory using a Sysmex XN9000 hematology analyzer (Sysmex America, Lincolnshire, Illinois)	126 patients	36 (27–52)	55%	42, 898 screened, 212 eligible, 72 with missing index test, 14 with no reference test or other issues.	Documented CO exposure and/or signs and symptoms of CO intoxication and/or SpCO >10% a triage screening. Both adult and pediatric patients were eligible.	Hbmet >1.6% as determined with pulse oximetry (SpHbmet). Patients with acrylic nails, painted fingernails, or fingernail deformities.	Diagnostic accuracy of SpCO testing for CO poisoning compared with BGA COHb testing (10% COHb cutoff)	Diagnostic accuracy of SpCO testing for CO poisoning compared with BGA COHb testing with a different cutoff (15% COHB)	10% COHb for the primary outcome, 15% for the secondary outcome

### Risk of bias

**Figure 2:**
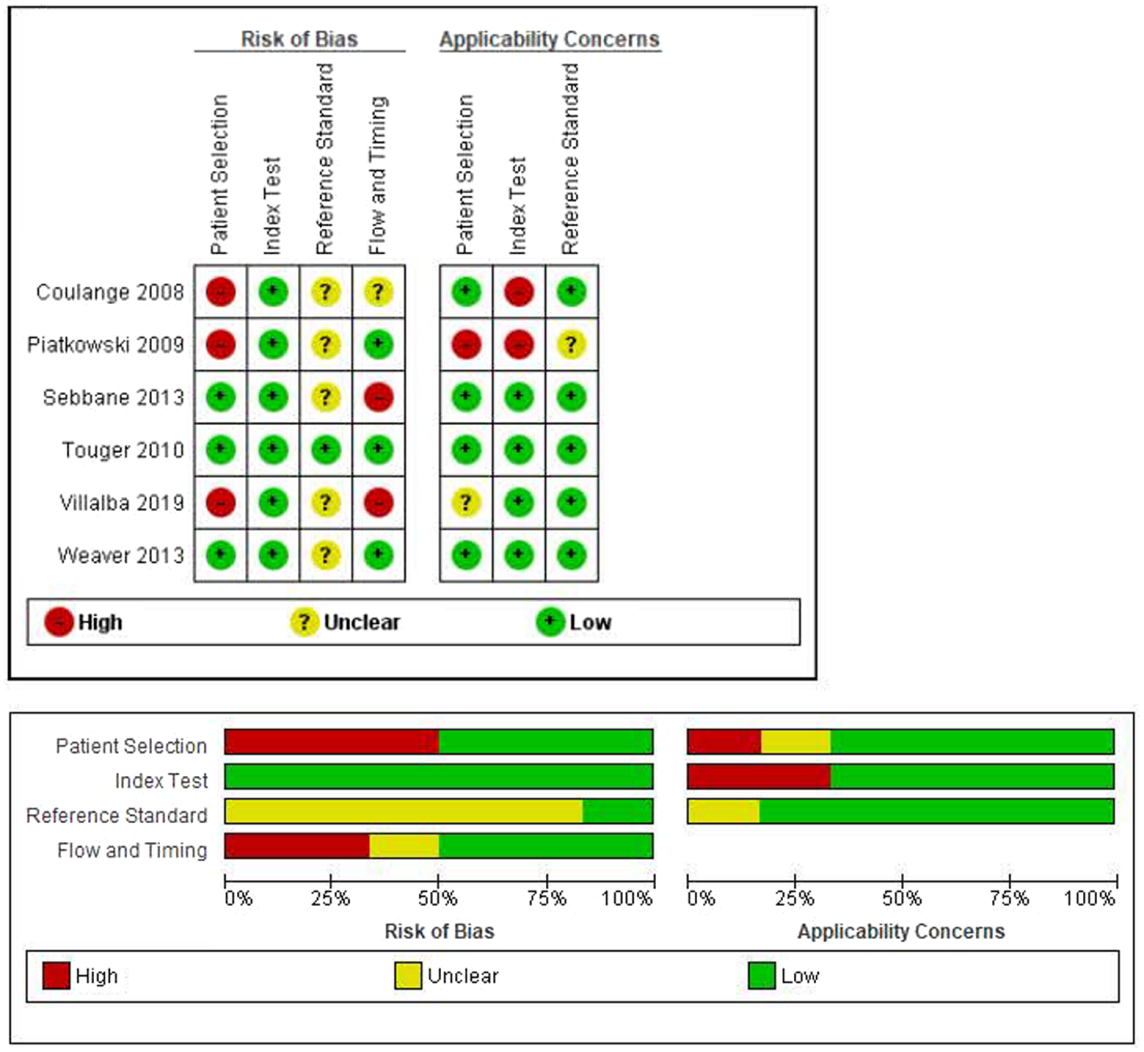
Evaluation of risk of bias and applicability concerns according to QUADAS-2 tool.

The risk of bias and applicability concerns evaluated according to QUADAS-2 is reported in Figure 2. Patient selection (lack of consecutive or random enrollment, inappropriate exclusions) and flow and timing (mostly due to the exclusion of patients from the analysis) were deemed to be at high risk of bias in three studies. While the reference standard was not deemed to be at high risk of bias in any study, it was unclear in six studies.

### Meta-analysis

**Figure 3:**
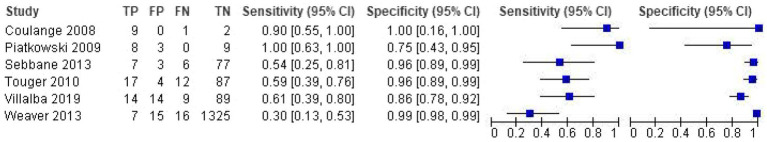
Forest plot of sensitivity and specificity of SpCO at a cut-off of 10%.

**Figure 4:**
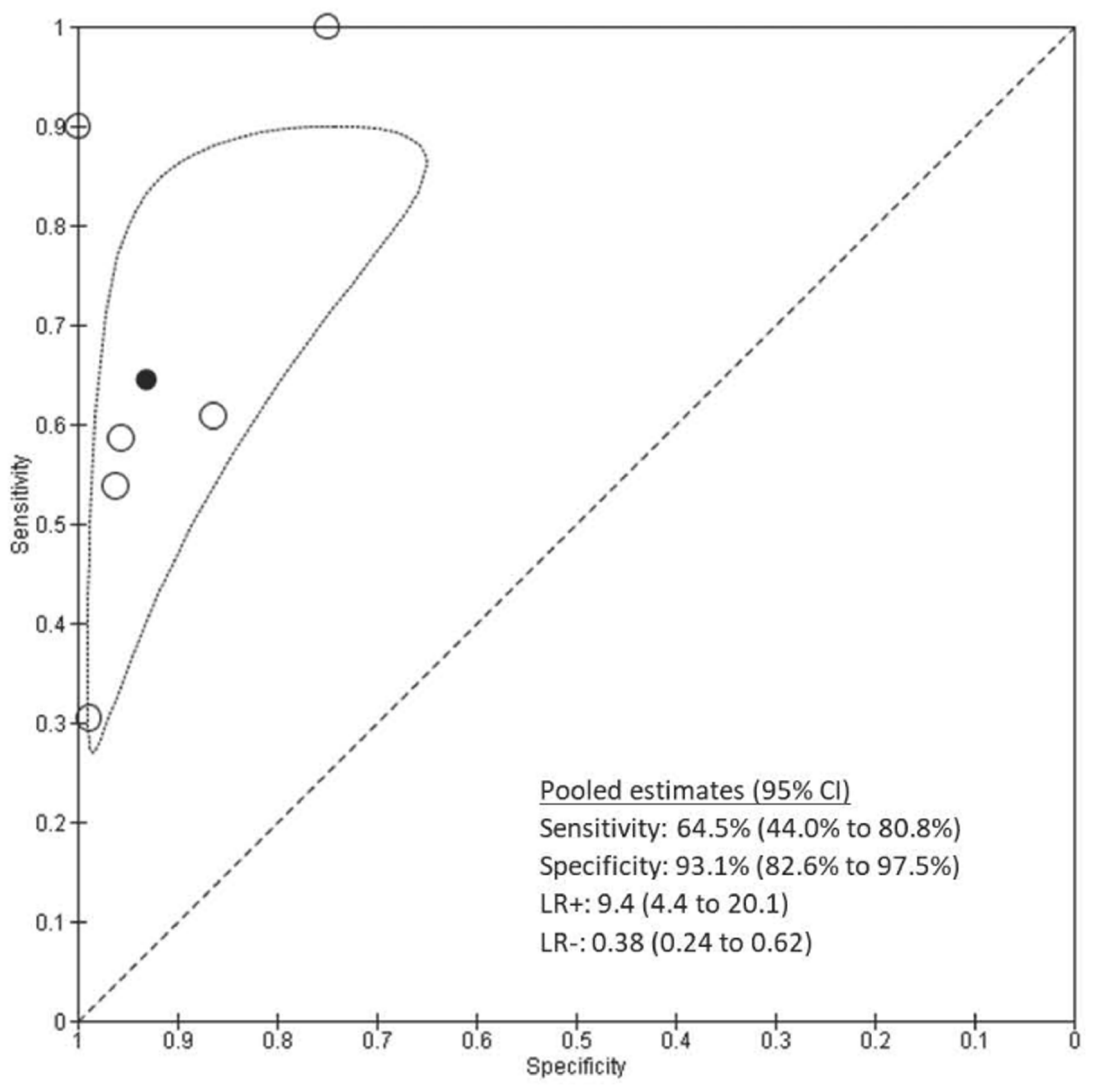
Plot of the studies in the ROC space. Circles represent individual studies. The solid black circle represents the pooled estimate of sensitivity and specificity. The dotted border represents 95% confidence region.

For the six studies included in the meta-analysis of SpCO at a 10% cutoff, sensitivity ranged from 30 to 100% and specificity ranged from 75 to 100% (Figure 3). The pooled sensitivity was 0.65 (95% CI 0.44–0.81), the pooled specificity was 0.93 (95% CI 0.83–0.98), the pooled LR+ was 9.4 (95% CI 4.4 to 20.1), and the pooled LR- was 0.38 (95% CI 0.24 to 0.62) (Figure 4).

## Discussion

The results of our study show that non-invasive SpCO at a COHb cutoff of 10% has high specificity (specificity 0.93; LR+ 9.4) but low sensitivity (sensitivity 0.65; LR- 0.38). These data suggest that SpCO is not sufficiently accurate to be used as a screening test in patients with suspected COP.

The ideal non-invasive method to triage patients with suspected COP in the ED should be highly sensitive. However, the sensitivity reported in our study is not sufficient to allow SpCO to be used as a screening test.

A COP diagnosis relies on clinical suspicion and increased levels of COHb. Blood COHb levels are usually considered normal, up to 10% in smokers and 5% in non-smokers (16). In our study, the defining CO cutoff for COP was 10%. This rather high upper limit was chosen because it is clinically relevant, it is outside of the CO physiological range for both smokers and non-smokers, and it was previously used to compare SpCO to blood COHb levels in a prospective observational study (15, 17). Lowering the COHb cutoff would likely increase SpCO sensitivity at the cost of specificity. While reducing the COHb cutoff to 5% could be a feasible strategy to improve the sensitivity of the test, it should be considered that the resulting decrease in specificity would result in a heightened rate of false positives and a consequent challenge in the management of resources. In addition, COHb levels may normally reach levels up to 10% in smokers, as compared to 2–3% in non-smokers, which further complicates diagnostic assessment (5). According to previous studies, each pack of cigarettes smoked a day increases COHb levels by approximately 2.5%, and in selected heavy smokers, COHb levels can also increase above 10% (17).

Importantly, the high LR+ obtained in our study suggests that SpCO could be useful to confirm a suspect COP in settings where BGA is not available. SpCO could help first responders rapidly recognize patients who need to be transported to a specialized hospital setting with hyperbaric treatment capability. A triage algorithm for COP based on SpCO was proposed by Hampson et al. in 2006 (18). In Italy, SpCO prehospital measurement has been explored in one district, with a reduction in the time elapsed from rescue to hyperbaric oxygen treatment (19). Similar results were observed in a group of patients in the US, in which SpCO measurement performed either at the scene by first responders or in the ED led to significantly shorter times for hyperbaric oxygen treatment, which could in turn lead to a clinical benefit (20).

In addition, on-site measurement of SpCO would be of high value in situations, such as mass casualty incidents (e.g., fires and explosions), in which the resources needed (such as helicopters and, in a severe crisis, even ambulances) to rapidly distribute the high number of patients to different facilities may be limited. The rapidity of use, the lack of necessity of invasive procedures (BGA), and the portability are the main advantages of the CO pulse oximeter. These features could make these devices particularly useful in disaster medicine. Implementing SpCO measurement within the initial triage procedure of patients involved in a fire would offer a quick estimate of the number of patients requiring hyperbaric treatment and therefore allow a timelier coordination with the emergency center and the receiving hospitals. In addition, a quick detection of COP with a SpCO device could be particularly useful to identify environments that could possibly be dangerous for rescuers and could be a valuable alternative to BGA in situations where it is not readily available, such as in disaster medicine. In a mass COP event that occurred in Switzerland, SpCO measurement was integrated with symptoms (e.g., transient loss of consciousness) and history (e.g., known pregnancy) in order to develop a rapid triage system that allowed for the identification of patients requiring transport to an HBO center, transport to a general hospital, or who could be treated on-site or directly discharged (21). SpCO measurement could also be of value in mass COP events following an abrupt power outage (e.g., as a result of storms, floods, and earthquakes) and the consequent use of improvised heating or burning devices (22, 23).

A recently published systematic review and meta-analysis evaluating SpCO accuracy to estimate blood COHb quantification reported a mean sensitivity of 0.77 [95% CI (0.66–0.85)] and a mean specificity of 0.83 [95% CI (0.74–0.89)] (24). Several reasons might explain the slightly higher sensitivity and lower specificity of SpCO observed in Papin’s study compared to ours. First, due to less stringent inclusion criteria, a higher number of articles with both observational and experimental designs were analyzed. Second, the COHb cutoff was not selected *a priori* and ranged from 5 to 23%. This wide range of COHb thresholds could have influenced SpCO diagnostic accuracy. However, even with these limitations, the meta-analysis results are similar to ours regarding the low sensitivity, while the reported specificity is much lower than ours. For these reasons, our conclusions are slightly different: they suggest that SpCO is currently an unreliable diagnostic tool and cannot be used as a substitute for blood COHb to identify COP cases, while we think that, in specific settings such as prehospital emergency medicine, SpCO could have some role.

### Study limitations

The most important limitation of our meta-analysis is the origin of the data. Our results might have been influenced by the small number of patients enrolled in most of the studies. Moreover, the report with the highest number of subjects (14) included only a minority of patients with proven COP. Of note, the low prevalence of COP might make enrollment of consecutive patients difficult, therefore exposing studies to possible biases.

Moreover, the choice of a single COHb cutoff for smokers and non-smokers could be a limitation since these two populations have different baseline COHb levels. In particular, the choice of a 10% cutoff may be inappropriate and too high in some special populations, such as pregnant women, children, and patients with chronic CO intoxication. Nonetheless, the available data do not allow an accurate distinction between these subgroups of patients. Further studies are needed to evaluate if setting distinct cutoffs in different patient populations could improve the diagnostic accuracy of SpCO.

## Conclusion

Our results show that SpCO cannot be used as a screening tool for COP in the ED due to its low sensitivity. Because of its high LR+, it would be interesting to evaluate if SpCO could have a role in the prehospital setting as a tool to quickly identify COP patients and prioritize their transport to specialized hospitals on larger samples with a prospective design.

## Data availability statement

The raw data supporting the conclusions of this article will be made available by the authors, without undue reservation.

## Author contributions

GR, EK, AG, FA, IP, RC, and Gcol did the search strategy extracted the data and quality assessment. GR, GCos, GCa, and FG wrote the first draft of the manuscript. All authors discuss the results and contribute in writing the final manuscript.
